# Chemical Characterization and Pharmacological Activity of *Byrsonima crassifolia* (L.) Kunth (Murici): Antinociceptive Effects, Edema Reduction, and Molecular Docking

**DOI:** 10.1002/cbdv.71661

**Published:** 2026-09-20

**Authors:** Lucas S. Frota, Sara I. C. G. Barbosa, Maria R. C. de Oiveira, Gabriela A. do Nascimento, Sacha A. A. R. Santos, Hamilton M. Ishiki, Francisco E. A. Magalhães, Adriana R. Campos, Selene M. de Morais

**Affiliations:** ^1^ Postgraduate Program in Biotechnology, Natural Products Chemistry Laboratory State University of Ceará Fortaleza CE Brazil; ^2^ Postgraduate Program in Nutrition and Health State University of Ceará Fortaleza CE Brazil; ^3^ Nutrition Course State University of Ceará Fortaleza CE Brazil; ^4^ Center For Education, Science and Technology in the Inhamuns Region State University of Ceará Sobral CE Brazil; ^5^ Center For Experimental Biology (NUBEX) University of Fortaleza (UNIFOR) Fortaleza CE Brazil

**Keywords:** antinociceptive activity, *Byrsonima crassifolia*, molecular docking, phenolic compounds, zebrafish

## Abstract

This study investigated the chemical composition, anti‐inflammatory and antinociceptive activities of murici leaf (ELM) and bark (EBM) extracts using adult zebrafish (*Danio rerio*). HPLC analysis identified rutin, isoquercitrin, and kaempferol‐3‐*O*‐rutinoside as the major phenolic compounds. Both extracts exhibited low toxicity and no sedative effects at the lowest tested doses. ELM showed abdominal antinociceptive activity mediated by TRPA1 and significantly reduced carrageenan‐induced abdominal edema. EBM inhibited both corneal (TRPV1) and abdominal (TRPA1) nociception, producing effects similar to morphine. Molecular docking corroborated the in vivo findings, revealing favorable interactions with TRPA1, COX‐2, and TRPV1, supported by stable hydrogen‐bond formation. The combined chemical, biological, and computational evidence highlights murici as a promising natural source of multifunctional phytotherapeutic agents with antinociceptive activity and potential anti‐inflammatory properties.

## Graphical abstract

Hydroethanolic extracts from *Byrsonima crassifolia* leaves and bark exhibit potent anti‐inflammatory and antinociceptive activities in zebrafish. Phenolic compounds, particularly kaempferol‐3‐O‐rutinoside, isoquercitrin, and rutin, target TRPA1, TRPV1, and COX‐2, integrating phytochemical, biological, and molecular docking evidence to support murici as a promising source of multifunctional phytotherapeutic agents.

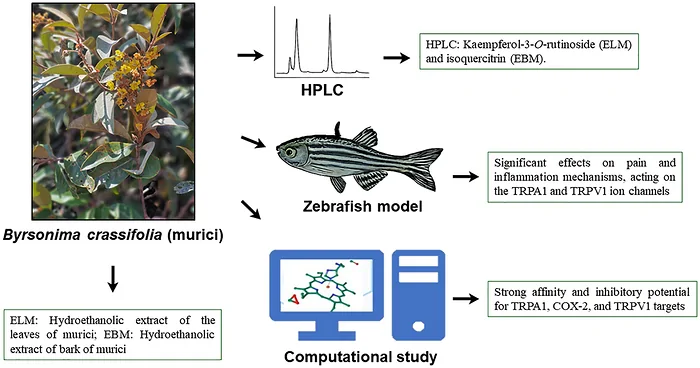

## 1 Introduction

Murici is the popular name assigned to several species of the genus *Byrsonima* (Malpighiaceae), a group of plants widely distributed throughout the Neotropical region, particularly in the Brazilian Cerrado, Caatinga, and Amazon biomes. Among them, *Byrsonima crassifolia* has attracted increasing scientific interest due to its extensive use in traditional medicine, where its fruits, leaves, bark, and seeds are employed for the treatment of inflammatory conditions, fever, gastrointestinal disorders, respiratory diseases, wound healing, and other ailments [1]. The growing search for natural products with therapeutic potential has reinforced the importance of investigating medicinal plants traditionally used by local communities, particularly those capable of providing bioactive molecules with fewer adverse effects than conventional anti‐inflammatory drugs [2].

Phytochemical investigations have demonstrated that *Byrsonima* species constitute an important source of phenolic compounds, especially flavonoids, phenolic acids, tannins, and carotenoids, metabolites widely recognized for their antioxidant, anti‐inflammatory, and analgesic properties [3]. Flavonoids such as rutin, isoquercitrin, quercetin, and kaempferol derivatives, frequently reported in species of this genus, have been shown to neutralize reactive oxygen species (ROS), chelate transition metals, and modulate intracellular signaling pathways involved in oxidative stress and inflammation [4]. Despite these findings, most pharmacological studies have focused on fruits or crude extracts, whereas the chemical composition and biological activities of leaves and stem bark remain comparatively underexplored, particularly regarding the integration of phytochemical characterization with experimental and computational approaches [5, 6].

Oxidative stress plays a pivotal role in the initiation and progression of inflammatory disorders by promoting excessive ROS production, lipid peroxidation, and activation of redox‐sensitive transcription factors such as nuclear factor‐kappa B (NF‐*κ*B) [7]. Consequently, the increased expression of pro‐inflammatory mediators, including cyclooxygenase‐2 (COX‐2), cytokines, and prostaglandins, contributes to nociceptor sensitization, tissue injury, and the maintenance of inflammatory pain. Therefore, natural antioxidants capable of restoring redox homeostasis may simultaneously attenuate oxidative damage and regulate inflammatory signaling pathways, representing promising candidates for the development of safer therapeutic agents [8].

In addition to oxidative stress modulation, the cholinergic anti‐inflammatory pathway has emerged as another important mechanism involved in the control of inflammatory responses. Acetylcholinesterase (AChE), the enzyme responsible for acetylcholine hydrolysis, regulates cholinergic neurotransmission and indirectly influences the activation of anti‐inflammatory signaling. Consequently, inhibition of AChE may increase acetylcholine availability, suppress pro‐inflammatory cytokine release, and reduce inflammatory responses [9]. Likewise, transient receptor potential channels, particularly TRPA1 and TRPV1, together with COX‐2, represent key molecular targets involved in nociception, neurogenic inflammation, and prostaglandin biosynthesis. Increasing evidence indicates that plant‐derived flavonoids are capable of interacting with these targets, providing molecular support for their analgesic and anti‐inflammatory effects [10, 11, 12].

The zebrafish (*Danio rerio*) has become a valuable experimental model for pharmacological screening because of its high genetic homology with humans, conserved inflammatory pathways, rapid development, and suitability for behavioral, toxicological, and mechanistic studies. Furthermore, zebrafish models enable the simultaneous evaluation of efficacy and safety while reducing experimental costs and complying with current principles for the ethical use of laboratory animals [13].

Based on the traditional medicinal use of *B. crassifolia* and the biological properties attributed to its phenolic constituents, we hypothesized that hydroethanolic extracts obtained from its leaves and stem bark exert antioxidant, antinociceptive, and anti‐inflammatory effects through the modulation of oxidative stress and inflammatory molecular targets. Therefore, the present study aimed to chemically characterize hydroethanolic extracts from the leaves and stem bark of *B. crassifolia*, investigate their antioxidant and antinociceptive activities in zebrafish models, and to evaluate their effects on carrageenan‐induced edema. Molecular docking analyses were performed to explore possible interactions with molecular targets associated with nociception and inflammatory responses. This multidisciplinary approach provides insights into the possible mechanisms underlying the pharmacological effects of this species, supports its traditional medicinal use, and contributes to the identification of bioactive compounds with potential therapeutic applications.

## 2 Results and Discussion

The hydroethanolic extracts obtained from murici leaves (ELM) and bark (EBM) exhibited distinct extraction yields and phytochemical profiles. ELM showed a yield of 18.72%, whereas EBM yielded 10.21%. Quantification of secondary metabolites revealed that ELM contained substantially higher concentrations of total phenolics (674.54 ± 1.48 mg GAE/g), flavonoids (412.53 ± 0.34 mg QE/g), and tannins (111.38 ± 3.72 mg TAE/g) than EBM, which presented 114.87 ± 0.47 mg GAE/g of phenolics, 59.42 ± 0.45 mg QE/g of flavonoids, and 22.24 ± 1.79 mg TAE/g of tannins (Table 1). The elevated concentration of phenolic constituents in ELM suggests a greater pharmacological potential, particularly considering the well‐established relationship between phenolic compounds and antioxidant activity [14].

**TABLE 1:** Quantification of metabolites of hydroethanolic extract of murici leaves and bark.

Samples	Total phenols (mg GAE/g)	Flavonoids (mg QE/g)	Total tannins (mg TAE/g)
ELM	674.54 ± 1.48	412.53 ± 0.34	111.38 ± 3.72
EBM	114.87 ± 0.47	59.42 ± 0.45	22.24 ± 1.79

EAG—Gallic acid equivalent in milligrams; EQ—Quercetin equivalent in milligrams; ELM—Hydroethanolic extract of murici leaves; EBM—Hydroethanolic extract of murici bark.

This phytochemical richness was reflected in the antioxidant assays (Table 2). ELM exhibited IC_50_ values of 7.10 ± 0.65 µg/mL and 10.40 ± 0.60 µg/mL against DPPH• and ABTS^+^•, respectively, statistically surpassing the antioxidant activity of the reference standard Trolox in both the DPPH (*q* = 15.61, *p* < 0.0001 vs. ELM; F2,6 = 186.8) and ABTS (*q* = 5.94, *p* < 0.05 vs. ELM; F2,6 = 1604) assays. Although EBM displayed lower activity, its IC_50_ values remained below 50 µg/mL, which is still classified as high antioxidant activity [15]. In addition, both extracts demonstrated strong acetylcholinesterase inhibitory activity, with IC_50_ values of 9.76 ± 0.51 µg/mL for ELM and 19.46 ± 0.45 µg/mL for EBM (Table 2), meeting the criteria for potent inhibition established by Santos et al. [16].

**TABLE 2:** Evaluation of the antioxidant and antiacetylcholinesterase activity of the hydroethanolic extract of murici leaves and bark.

Samples	IC_50_ DPPH● (µg/mL)	IC_50_ ABTS^+^● (µg/mL)	IC_50_ AChE (µg/mL)
Trolox (+)	12.35 ± 0.05^b^	12.13 ± 0.07^b^	**—**
Galantamine (+)	**—**	**—**	5.82 ± 0.02^a^
ELM	7.10 ± 0.65^a^	10.40 ± 0.60^a^	9.76 ± 0.51^b^
EBM	16.26 ± 0.77^c^	31.39 ± 0.63^c^	19.46 ± 0.45^c^

IC_50_ ‐ Median inhibitory concentration for 50%; Trolox ‐ 6‐hydroxy‐2,5,7,8‐tetramethylchromano‐2‐carboxylic acid; ELM—Hydroethanolic extract of murici leaves; EBM—Hydroethanolic extract of murici bark. Different lowercase letters indicate statistically significant differences within the group (*p* < 0.05, ANOVA followed by Tukey's test).

The inhibition of acetylcholinesterase is particularly relevant because increased AChE activity has been associated with chronic inflammatory conditions. Enhanced degradation of acetylcholine compromises the cholinergic anti‐inflammatory pathway, resulting in increased production of pro‐inflammatory cytokines such as TNF‐α and IL‐1β and activation of NF‐κB signaling [17]. Therefore, the combined antioxidant and anti‐AChE activities observed for the extracts may contribute to the modulation of inflammatory processes.

HPLC analysis revealed marked differences in the phenolic composition of the extracts (Table 3). Kaempferol‐3‐*O*‐rutinoside was identified as the major constituent of ELM (59.71 ± 0.48 mg/g), followed by isoquercitrin (22.02 ± 0.11 mg/g) and rutin (2.93 ± 0.25 mg/g). In contrast, isoquercitrin was the predominant flavonoid in EBM (13.72 ± 0.18 mg/g). These compounds have been extensively associated with antioxidant, anti‐inflammatory, and antinociceptive activities. Kaempferol‐3‐*O*‐rutinoside has been described as a potent anti‐inflammatory and antinociceptive flavonoid with effects comparable to morphine in experimental models [18, 19]. Isoquercitrin exhibits antioxidant, neuroprotective, anti‐inflammatory, and antiacetylcholinesterase activities [20], while rutin is recognized for its antioxidant and vasoprotective properties [21].

**TABLE 3:** HPLC quantification of phenolic compounds in hydroethanolic extract of murici leaves and bark.

Constituents	R_t_ (min)	Concentration (mg/g of extract)	
		ELM	EBM
**Phenolic acids**			
Gallic acid	3.56	0.95 ± 0.23	0.34 ± 0.05
**Flavonoids**			
Rutin	6.70	2.93 ± 0.25	0.21 ± 0.15
Isoquercitrin	7.90	22.02 ± 0.11	13.72 ± 0.18
Kaempferol‐3‐*O*‐rutinoside	10.48	59.71 ± 0.48	—
Quercetin	23.03	1.99 ± 0.41	0.25 ± 0.18

R_t_, Retention time; ELM, Hydroethanolic extract of the leaves of murici; EBM, Hydroethanolic extract of bark of murici.

**FIGURE 1:**
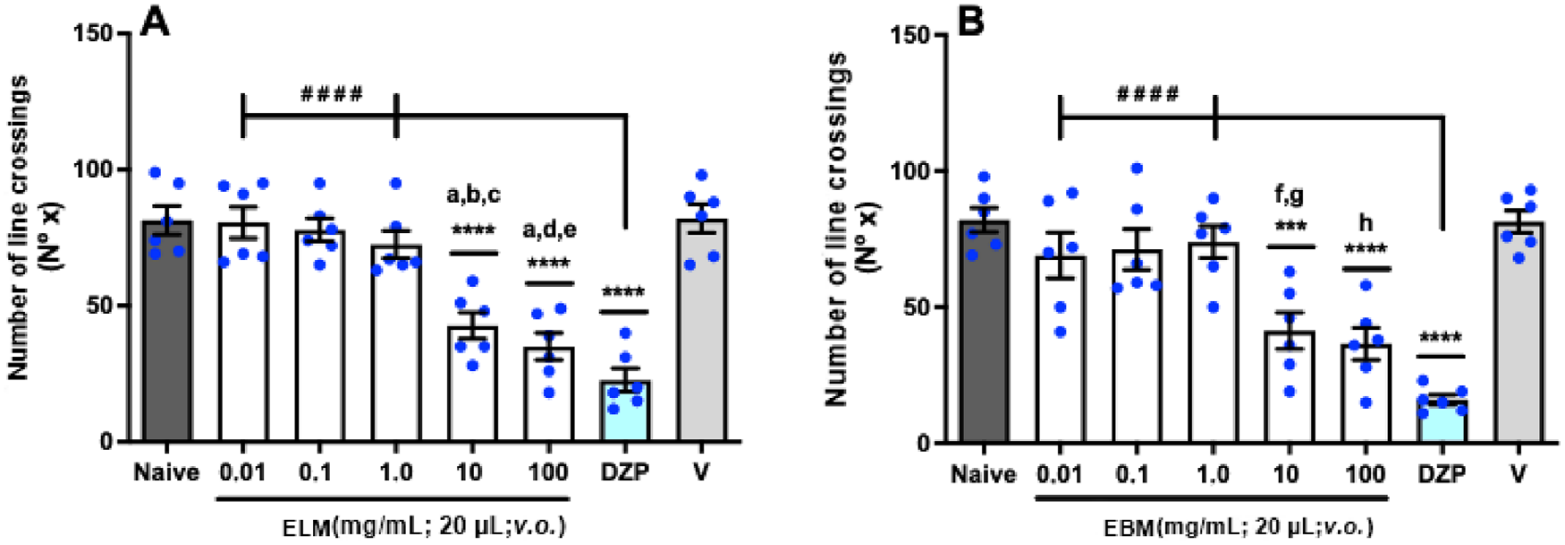
Effect of (A) ELM and (B) EBM on the locomotor activity in adult zebrafish (ZFa), analyzed in the Open Field Test (0–5 min). Naive – Untreated animals. DZP – Diazepam (Sedative control; 10 mg/mL; 20 µL; p.o.). V – Vehicle (DMSO 3%; 20 µL; p.o.). Values represent the mean ± standard error of the mean (S.E.M.) for 6 animals per group. ANOVA followed by Tukey's test (****p < 0.0001 vs. Naive or Vehicle; ^####^ *p* < 0.0001 vs. DZP; ^a^ *p* < 0.0001 vs. 0.01 mg/mL EFM; ^b^ *p* < 0.001 vs. 0.1 mg/mL EFM; ^c^ *p* < 0.01 vs. 1.0 mg/mL EFM; ^d^ *p* < 0.0001 vs. 0.1 mg/mL EFM; ^e^ *p* < 0.001 vs. 1.0 mg/mL EFM; ^g^ *p* < 0.05 vs. 0.01 or 0.1 mg/mL EGM; ^h^ *p* < 0.01 vs. 1.0 mg/mL EGM; ^i^ *p* < 0.01 vs. 0.01, 0.1, or 1.0 mg/mL EGM).

In the acute toxicity assay, neither ELM nor EBM induced mortality in adult zebrafish during the 96‐h observation period, even at the highest concentration tested (100 mg/mL), indicating LC_50_ values greater than 100 mg/mL and suggesting low toxicity. In the locomotor activity test (Open Field Test), the three lowest doses of ELM showed no sedative effect, as the ZFa treated with ELM (0.01, 0.1 or 1.0 mg/mL; 20 µL; p.o.) exhibited a significantly different effect [(*q* = 11.58; *q* = 11.04; *q* = 9.975) *p* < 0.0001 vs. DZP] compared to animals treated with diazepam (DZP; 10 mg/mL; 20 µL; p.o.), used as the sedative control (DZP: *q* = 11.74, *p* < 0.0001 vs. Naïve or *q* = 11.88, *p* < 0.0001 vs. Vehicle). The locomotor activity results are shown in Figure 1A (F_7_,_40_ = 23.62). Similarly, the three lowest doses of EBM did not show sedative effects, as the ZFa treated with EBM (0.01, 0.1 or 1.0 mg/mL; 20 µL; p.o.) exhibited a significantly different effect [(*q* = 8.963; *q* = 9.330; *q* = 9.809) *p* < 0.0001 vs. DZP] compared to animals treated with diazepam (DZP; 10 mg/mL; 20 µL; p.o.), used as the sedative control (DZP: *q* = 11.16, *p* < 0.0001 vs. Naïve or *q* = 11.05, *p* < 0.0001 vs. Vehicle). The locomotor activity results are shown in Figure 1B (F_7_,_40_ = 17.03).

**FIGURE 2:**
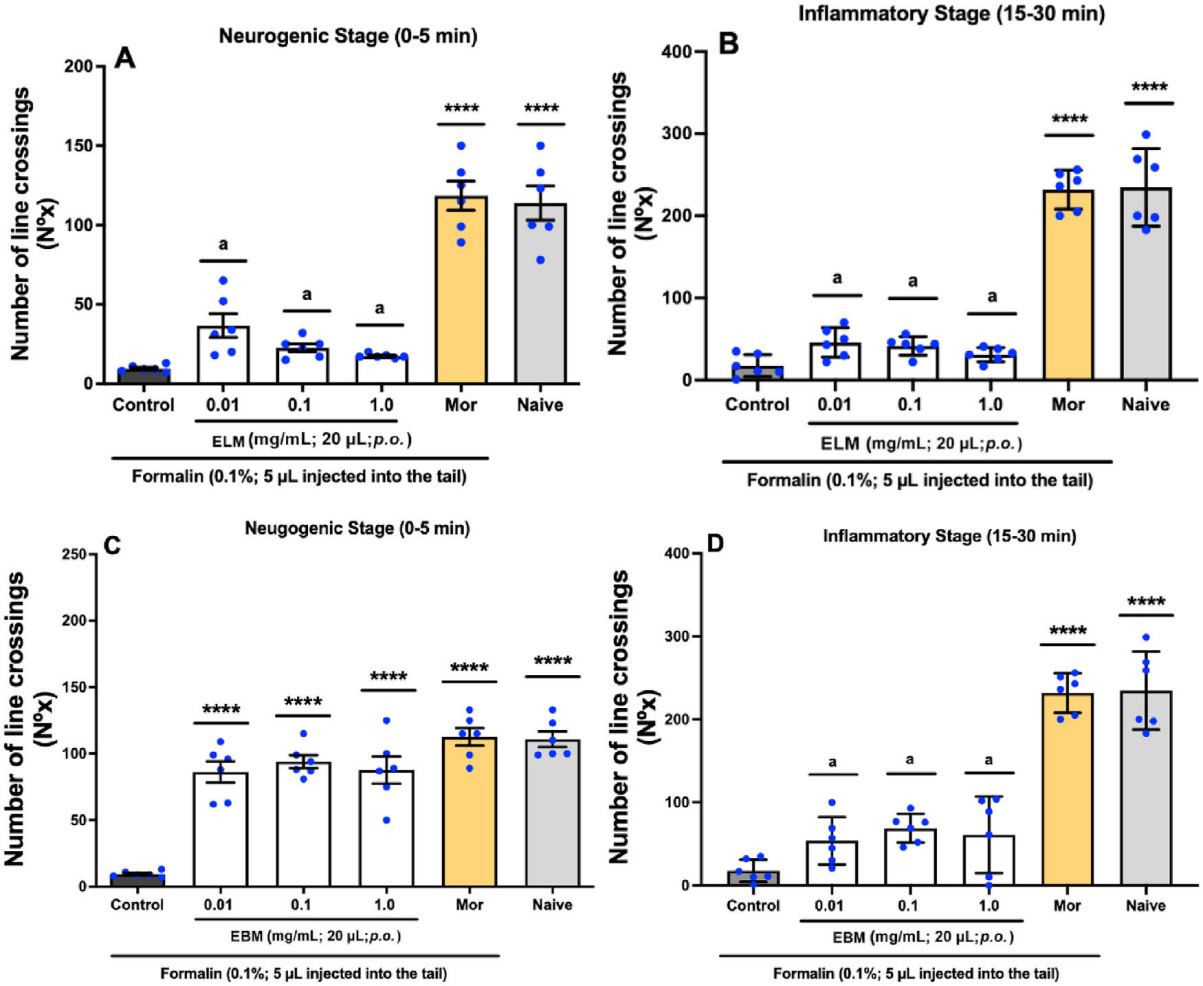
Effect of ELM (A and B) and EBM (C and D) on nociceptive behavior in adult zebrafish (ZFa) induced by formalin, analyzed in both phases of the test. Control – Vehicle (DMSO 3%; 20 µL; p.o.). Mor – Morphine (Antinociceptive control; 0.2 mg/mL; 20 µL; i.p.). Naive – Untreated animals. Values represent the mean ± standard error of the mean (S.E.M.) for 6 animals per group. ANOVA followed by Tukey's test (****p < 0.0001 vs. Control; ^a^ *p* < 0.0001 vs. Mor or Naive).

The ELM did not inhibit the nociceptive behavior in ZFa induced by formalin (0.1%) in either phase of the test, as ZFa treated with EFM (0.01, 0.1 or 1.0 mg/mL; 20 µL; p.o.) showed a response significantly [Figure 2A: (*q* = 4.104; *q* = 1.989; *q* = 1.208) *p* < 0.05 vs. Control; Figure 2B: (*q* = 2.858; *q* = 2.436; *q* = 1.353) *p* < 0.05 vs. Control] similar to the Control group (DMSO 3%; 20 µL; p.o.). This effect of ELM was significantly [Figure 2A: (*q* = 12.36; *q* = 14.48; *q* = 15.26) *p* < 0.0001; Figure 2B: (*q* = 18.88; *q* = 19.30; *q* = 20.38) *p* < 0.0001 vs. Morphine] different from that observed in animals treated with morphine (Mor; 0.2 mg/mL; 20 µL; i.p.), used as the antinociceptive control [Figure 2A: *q* = 16.47, *p* < 0.0001 vs. Control or *q* = 0.7050, *p* > 0.05 vs. Naive; Figure 2B]. The results are illustrated in Figure 2A (F_5_,_30_ = 56.27) and Figure 2B (F_5_,_30_ = 110.0). On the other hand, the EBM inhibited nociceptive behavior in ZFa induced by formalin (0.1%) only in the neurogenic phase of the test (Figure 2C), as ZFa treated with EBM (0.01, 0.1 or 1.0 mg/mL; 20 µL; p.o.) showed an effect significantly [(*q* = 3.947; *q* = 2.780; *q* = 3.723) *p* < 0.05 vs. Morphine] similar to that of the animals treated with morphine (Mor; 0.2 mg/mL; 20 µL; i.p.), used as the antinociceptive control (Figure 2C: *q* = 15.36, *p* < 0.0001 vs. Control). The results are illustrated in Figure 2C (F_5_,_30_ = 31.98) and Figure 2D (F_5_,_30_ = 53.48).

**FIGURE 3:**
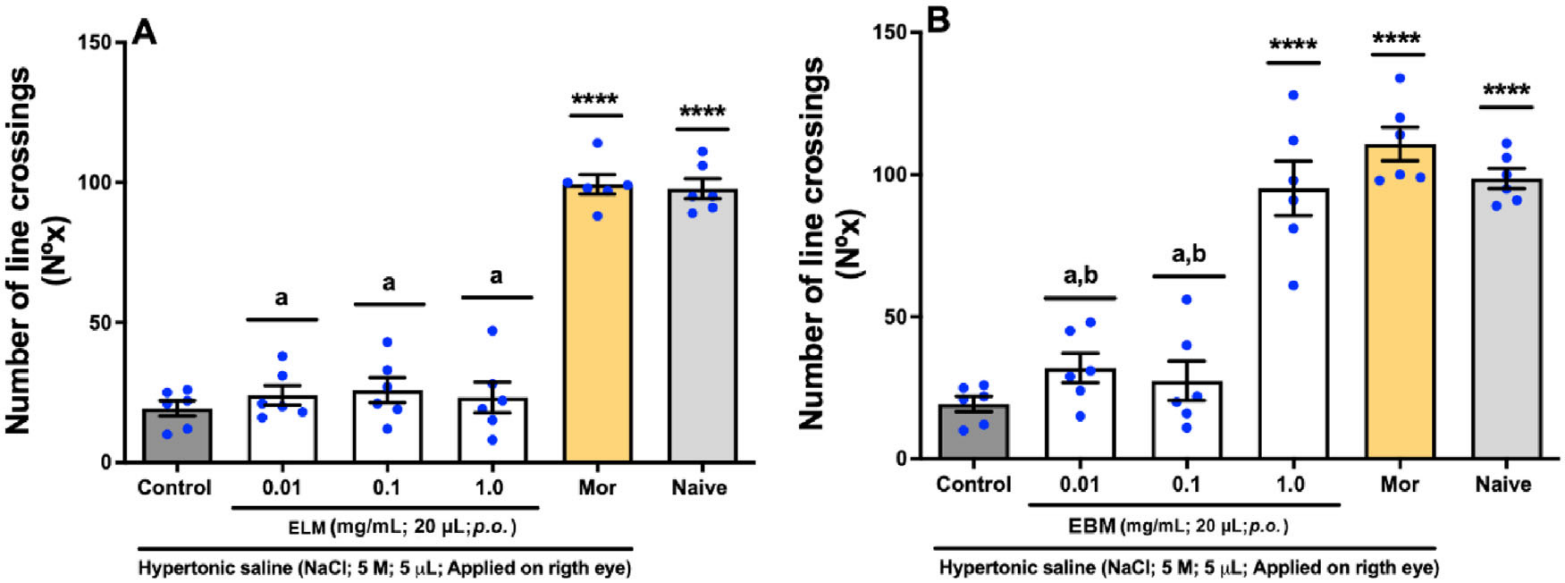
Effect of ELM (A) and EBM (B) on corneal nociceptive behavior in adult zebrafish (ZFa) induced by hypertonic saline, analyzed over 5 min. Control – Vehicle (DMSO 3%; 20 µL; p.o.). Mor – Morphine (antinociceptive control; 0.4 mg/mL; 20 µL; i.p.). Naive – Untreated animals. Values represent the mean ± standard error of the mean (S.E.M.) for 6 animals per group. ANOVA followed by Tukey's test (****p < 0.0001 vs. Control; ^a^ *p* < 0.0001 vs. Mor or Naive; ^b^ *p* < 0.0001 vs. EBM 1.0 mg/mL).

**FIGURE 4:**
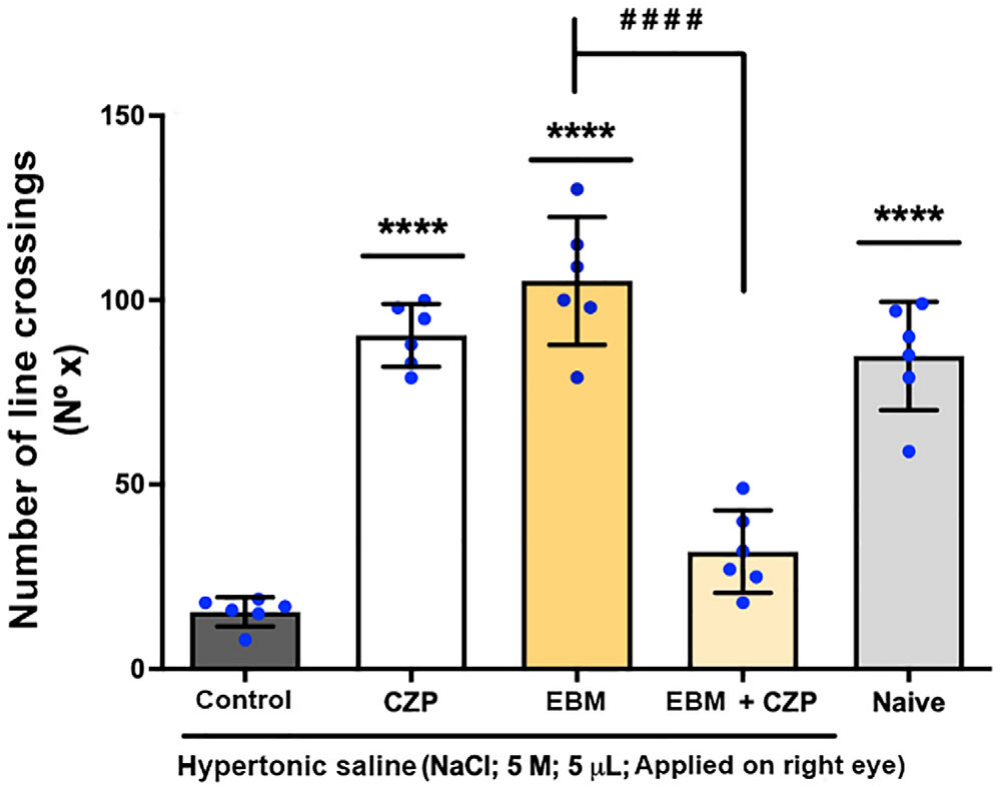
Effect of capsazepine (CZP; TRPV1 antagonist; 0.5 mg/mL; 20 µL; i.p.) on the corneal antinociceptive effect of EBM (1.0 mg/mL; 20 µL; p.o.) in adult zebrafish (ZFa), analyzed from 0 to 5 min. Control – Vehicle (3% DMSO; 20 µL; p.o.). Naive – Untreated animals. Values represent the mean ± standard error of the mean (S.E.M.) for 6 animals per group. ANOVA followed by Tukey's test (^****^ *p* < 0.0001 vs. Control; ^####^ *p* < 0.0001 vs. EBM).

The ELM did not inhibit corneal nociceptive behavior in ZFa induced by hypertonic saline (NaCl, 5 M, 5 µL applied to the right eye), as ZFa treated with ELM (0.01, 0.1 or 1.0 mg/mL; 20 µL; p.o.) showed an effect significantly [(*q* = 1.174; *q* = 1.635; *q* = 0.9642) *p* < 0.05 vs. Control] similar to that of the Control group (DMSO 3%; 20 µL; p.o.). This effect of ELM was significantly [(*q* = 18.95; *q* = 18.49; *q* = 19.16) *p* < 0.0001 vs. Morphine] different from the effect observed in animals treated with morphine (Mor; 0.4 mg/mL; 20 µL; i.p.), used as the antinociceptive control (*q* = 20.12, *p* < 0.0001 vs. Control or *q* = 0.3773, *p* > 0.05 vs. Naive). The results are illustrated in Figure 3A (F_5_,_30_ = 96.47). The EBM inhibited corneal nociceptive behavior in ZFa induced by hypertonic saline (NaCl, 5 M, 5 µL applied to the right eye), as ZFa treated with EGM (1.0 mg/mL; 20 µL; p.o.) showed a significantly different effect (*q* = 12.47, *p* < 0.0001 vs. Control) from that of the Control group (DMSO 3%; 20 µL; p.o.). This effect of EBM was significantly (*q* = 2.576, *p* < 0.05 vs. Morphine) similar to that of the animals treated with morphine (Mor; 0.4 mg/mL; 20 µL; i.p.), used as the antinociceptive control (*q* = 15.05, *p* < 0.0001 vs. Control or *q* = 0.2001, *p* > 0.05 vs. Naive). The results are illustrated in Figure 3B (F_5_,_30_ = 47.15). Importantly, pretreatment with capsazepine abolished the antinociceptive effect of EBM (Figure 4), confirming the participation of TRPV1 receptors in the observed response. This result is consistent with the known involvement of TRPV1 channels in corneal nociception and inflammatory pain.

Capsazepine significantly prevented (*q* = 11.93, *p* < 0.0001 vs. EBM + CZP) the corneal antinociceptive effect of EBM in adult zebrafish (ZFa), as shown in Figure 4 (F_4_,_25_ = 64.12).

**FIGURE 5:**
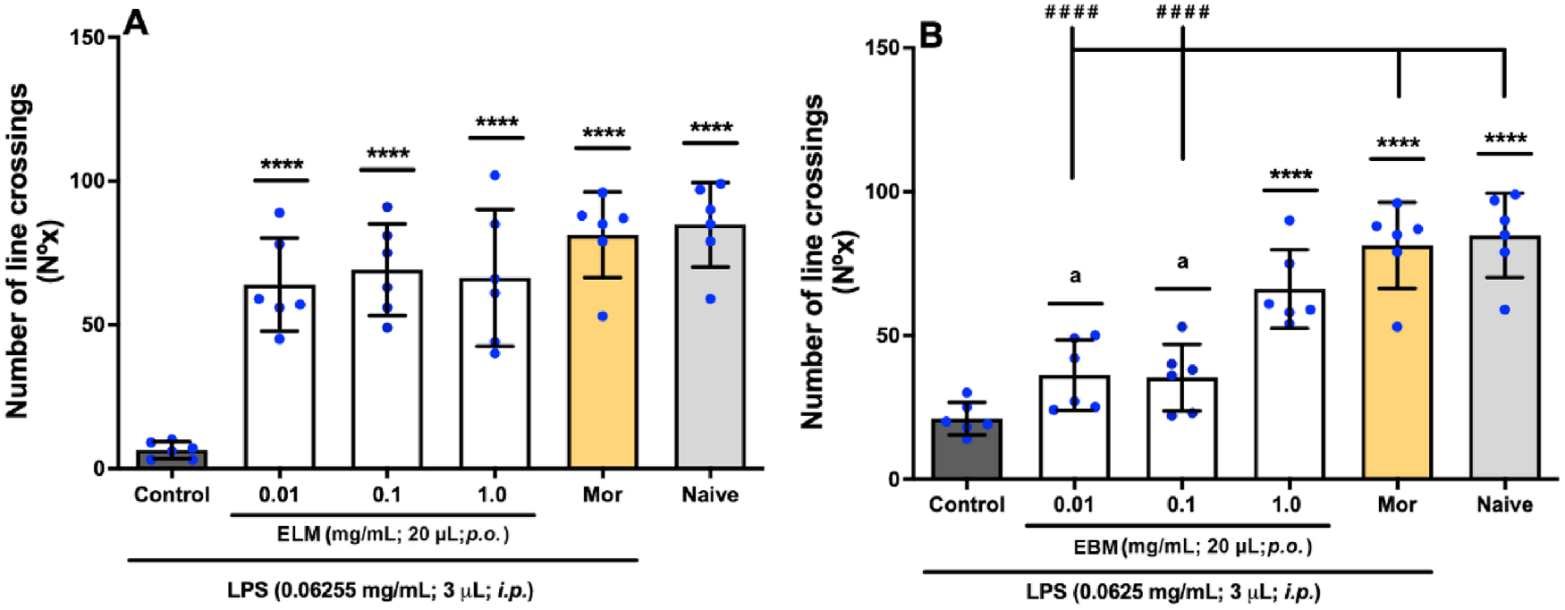
Effect of ELM (A) and EBM (B) on abdominal nociceptive behavior in adult zebrafish (ZFa) induced by LPS (TRPA1 agonist; 0.0625 mg/mL; 3.0 µL; i.p.), analyzed over 5 min. Control – Vehicle (3% DMSO; 20 µL; p.o.). Mor – Morphine (antinociceptive control; 0.2 mg/mL; 5.0 µL; i.p.). Naive – Untreated animals. Values represent the mean ± standard error of the mean (S.E.M.) for 6 animals per group. ANOVA followed by Tukey's test (^****^ *p* < 0.0001 vs. Control; ^a^ *p* < 0.0001 vs. EBM 1.0 mg/mL).

The ELM inhibited the abdominal nociceptive behavior in adult zebrafish (ZFa) induced by LPS (TRPA1 agonist; 0.0625 mg/mL; 3.0 µL; i.p.), as ZFa treated with ELM (0.01, 0.1, or 1.0 mg/mL; 20 µL; p.o.) showed a significantly [(*q* = 8.838; *q* = 9.630; *q* = 9.195) *p* < 0.0001 vs. Control] different effect compared to the Control group (3% DMSO; 20 µL; p.o.). This effect of ELM was significantly [(*q* = 2.625; *q* = 1.865; *q* = 2.299) *p* < 0.05 vs. Morphine] similar to that of animals treated with morphine (Mor; 0.2 mg/mL; 5.0 µL; i.p.), used as an abdominal antinociceptive control (*q* = 11.49, *p* < 0.0001 vs. Control or *q* = 0.5364, *p* > 0.05 vs. Naive). The results are shown in Figure 5A (F_5_,_30_ = 19.11). The EBM also inhibited the abdominal nociceptive behavior in ZFa induced by LPS (TRPA1 agonist; 0.0625 mg/mL; 3.0 µL; i.p.), as ZFa treated with EBM (1.0 mg/mL; 20 µL; p.o.) showed a significantly (*q* = 8.832, *p* < 0.0001 vs. Control) different effect compared to the Control (3% DMSO; 20 µL; p.o.). This effect of EBM was significantly (*q* = 2.966, *p* < 0.05 vs. Morphine) similar to that of animals treated with morphine (Mor; 0.2 mg/mL; 5.0 µL; i.p.), used as an abdominal antinociceptive control (*q* = 11.80, *p* < 0.0001 vs. Control or *q* = 0.6844, *p* > 0.05 vs. Naive). The results are shown in Figure 5B (F_5_,_30_ = 27.54).

**FIGURE 6:**
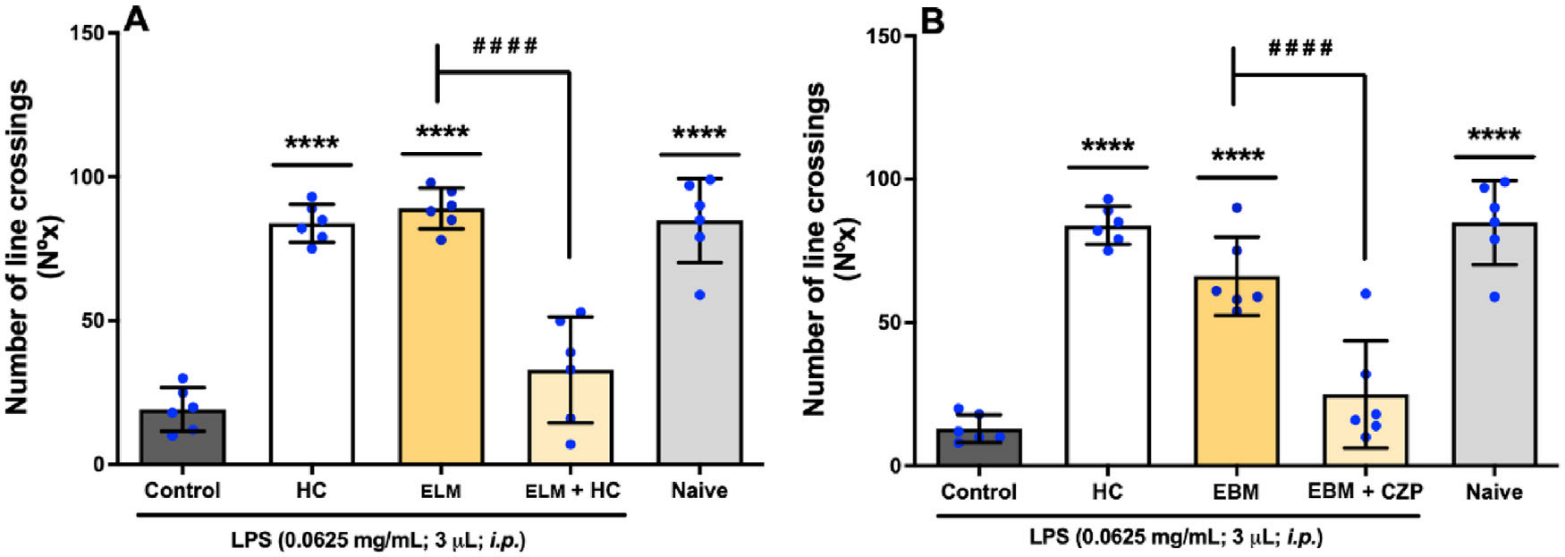
Effect of HC (HC‐030031; TRPA1 antagonist; 0.1 mg/mL; 5.0 µL; i.p.) on the abdominal antinociceptive effect of (A) ELM (0.01 mg/mL; 20 µL; p.o.) and (B) EBM (1.0 mg/mL; 20 µL; p.o.) in adult zebrafish (ZFa). Abdominal nociception was induced by LPS (TRPA1 agonist; 0.0625 mg/mL; 3.0 µL; i.p.) and analyzed for 5 min. Control – Vehicle (DMSO 3%; 20 µL; p.o.). Naive – Untreated animals. Values represent the mean ± standard error of the mean (S.E.M.) for 6 animals per group. ANOVA followed by Tukey's test (^**^ *p* < 0.0001 vs. Control; ^####^ *p* < 0.0001 vs. ELM or EBM).

HC (HC‐030031; TRPA1 antagonist; 0.1 mg/mL; 5.0 µL; i.p.) significantly prevented (ELM: *q* = 10.48, *p* < 0.0001 vs. ELM + CZP; EBM: *q* = 11.25, *p* < 0.0001 vs. EBM + CZP) the abdominal antinociceptive effect of ELM and EBM in ZFa, as shown in Figure 6A (F_4,25_ = 46.79) and Figure 6B (F_4,25_ = 41.24), respectively.

**FIGURE 7:**
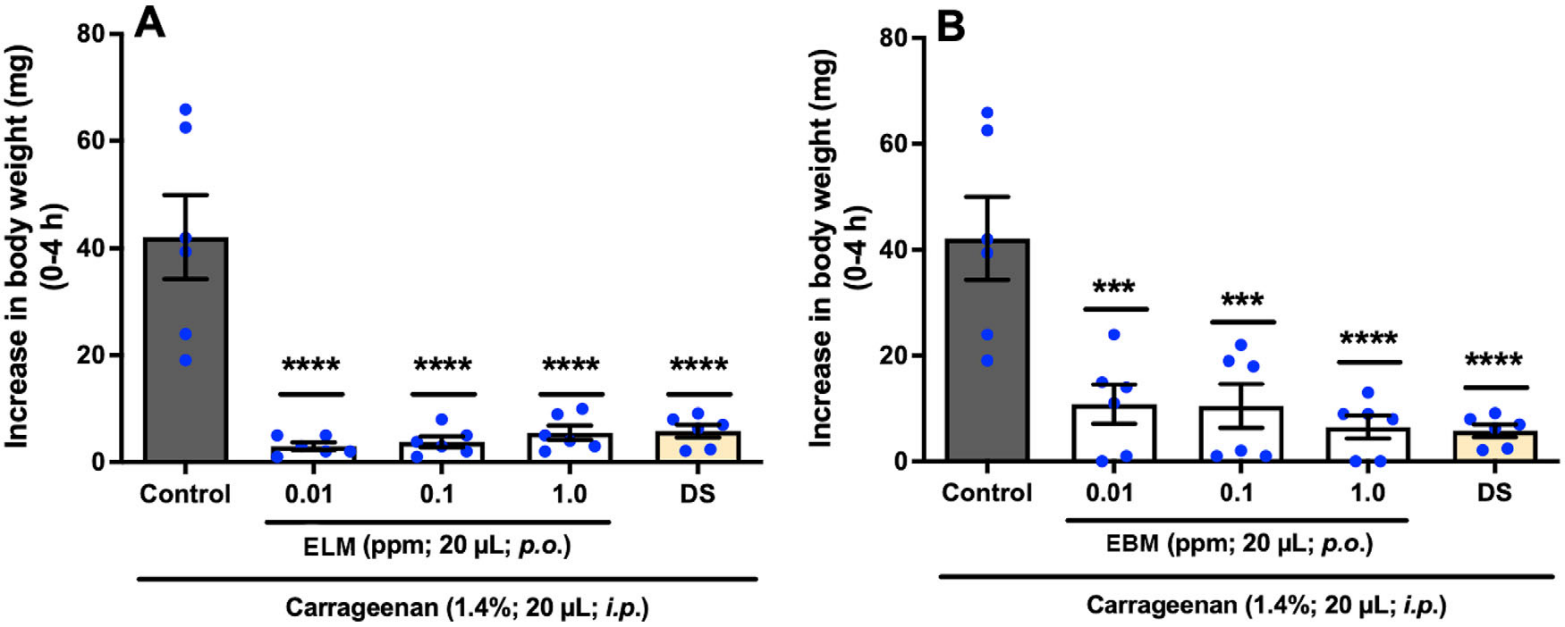
Effect of ELM (A) and EBM (B) on abdominal edema in adult zebrafish (ZFa) induced by carrageenan (1.4%; 20 µL; i.p.), analyzed individually (0–4 h). Control – Vehicle (DMSO 3%; 20 µL; p.o.). DS – Sodium diclofenac (anti‐inflammatory control; 2.5 mg/mL; 20 µL; p.o.). Values represent the mean ± standard error of the mean (S.E.M.) for 6 animals per group. ANOVA followed by Tukey's test (^***^ *p* < 0.001; ^**^ *p* < 0.0001 vs. Control).

The present study demonstrated significant antinociceptive activity of the murici extracts in zebrafish. In addition, the extracts significantly reduced carrageenan‐induced abdominal edema, indicating a potential effect on inflammatory processes. Pretreatment with ELM (0.01, 0.1 or 1.0 mg/mL; 20 µL; p.o.) significantly reduced abdominal edema in adult zebrafish (ZFa) induced by carrageenan (1.4%; 20 µL; i.p.) [(*q* = 10.76; *q* = 10.54; *q* = 10.07), *p* < 0.0001 vs. Control], differing from the Control group (DMSO 3%; 20 µL; p.o.). This effect of ELM was significantly [(*q* = 1.758; *q* = 2.177; *q* = 0.9767), *p* < 0.05 vs. DS] similar to that of animals treated with sodium diclofenac (DS; 2.5 mg/mL; 20 µL; p.o.), used as an anti‐inflammatory control (*q* = 9.982, *p* < 0.0001 vs. Control). The results are illustrated in Figure 7A (F_4_,_25_ = 21.47). Pretreatment with EBM (0.01, 0.1 or 1.0 mg/mL; 20 µL; p.o.) significantly reduced abdominal edema in ZFa induced by carrageenan (1.4%; 20 µL; i.p.) [(*q* = 7.051; *q* = 7.126, *p* < 0.001; *q* = 8.027, *p* < 0.0001 vs. Control)], differing from the Control (DMSO 3%; 20 µL; p.o.). This effect of EBM was significant [(*q* = 1.130; *q* = 1.054; *q* = 0.1539), *p* < 0.05 vs. DS], similar to that of animals treated with sodium diclofenac (DS; 2.5 mg/mL; 20 µL; p.o.), used as the anti‐inflammatory control (*q* = 8.180, *p* < 0.0001 vs. Control). The results are illustrated in Figure 7B (F_4_,_25_ = 11.80). The reduction in carrageenan‐induced edema observed in zebrafish is consistent with an effect on inflammatory responses. However, because no inflammatory mediators or signaling pathways were evaluated, these findings should be interpreted as functional evidence of edema reduction rather than direct mechanistic evidence of anti‐inflammatory activity.

To investigate the molecular basis of these effects, docking studies were performed against TRPA1, COX‐2, and TRPV1 receptors. For TRPA1, rutin exhibited the most favorable binding energy (−10.06 kcal/mol), surpassing the reference antagonist HC‐030031 (−9.77 kcal/mol) (Table 4). Isoquercitrin (−6.28 kcal/mol), kaempferol‐3‐O‐rutinoside (−6.31 kcal/mol), and quercetin (−6.32 kcal/mol) also interacted with residues involved in receptor modulation. Hydrophobic interactions with LEU708, TRP711, GLU854, ARG975, and GLN979, together with hydrogen bonds involving GLU854, MET978, and HIS983, contributed to ligand stabilization within the binding pocket.

**TABLE 4:** Interaction energy values between the TRPA1 receptor and each of the studied ligands, in kcal/mol, and the amino acid residues of the active site interacting with them.

Ligand	Energy (kcal/mol)	Interactions
HC‐030031 (+)	−9.77	HI: ILE858, ARG975, MET978; HB: ARG852, GLU854, HIS983; PS: TRP711; HI: GLU854
Gallic Acid	−4.33	HB: GLN851, GLU854, GLN979; SB: ARG975; HI: GLU854, GLN979
Isoquercitrin	−6.28	HB: GLU854, GLN979; PS: TRP711; HI: LEU708, GLU854, GLN979
Kaempferol‐3‐*O*‐rutinoside	−6.31	HB: GLU854, MET978, GLN979; PS: TRP711; HI: TRP711, GLU854, GLN979
Quercetin	−6.32	HB: GLU854, MET978, GLN979; HI: GLN979
Rutin	−10.06	HB: TRP711, GLU854, ARG975, HIS983; PS: TRP711

HI, hydrophobic interactions; HB, hydrogen bonds; PS, pi‐stacking; SB, salt bridges.

The relevance of these interactions is reinforced by the fact that HC‐030031 acts through an allosteric site located near the S5–S6 transmembrane region, preventing channel opening [10]. Therefore, the occupation of similar regions by rutin and other flavonoids suggests a comparable inhibitory mechanism, supporting the TRPA1‐mediated antinociceptive activity observed in vivo.

Molecular docking revealed favorable binding affinities between major phytochemicals and COX‐2, suggesting that modulation of this enzyme may contribute to the reduction of carrageenan‐induced edema. Rutin (−13.87 kcal/mol), isoquercitrin (−10.63 kcal/mol), and kaempferol‐3‐*O*‐rutinoside (−8.48 kcal/mol) exhibited interaction energies equal to or more favorable than diclofenac (−8.28 kcal/mol) (Table 5). These compounds established multiple hydrogen bonds and hydrophobic interactions with key catalytic residues such as ARG120, TYR355, PHE518, and SER530. Interestingly, rutin and isoquercitrin displayed stronger affinities than several flavonoids recently proposed as COX‐2 inhibitors, including cudraflavone A (−10.19 kcal/mol) [22].

**TABLE 5:** Interaction energy values between the COX‐2 receptor and each of the studied ligands, in kcal/mol, and the active site amino acid residues interacting with them.

Ligand	Energy (kcal/mol)	Interactions
Diclofenaco (+)	−8.28	HI: VAL349, LEU352, TYR385, TRP387, PHE518, VAL523, ALA527; HB: TYR355, VAL523 HALB: MET522; SB: ARG120
Gallic Acid	−4.53	HI: LEU352, TRP387; HB: TYR348, TYR385, MET522, GLY526, ALA527, SER530
Isoquercitrin	−10.63	HI: LEU352, TRP387, ILE517, PHE518; HB: ARG120, GLN192, TYR355, ILE517, PHE518, GLY526, SER530
Kaempferol‐3‐*O*‐rutinoside	−8.48	HI: PHE205, VAL349, LEU352, PHE381, LEU384, TYR385, TRP387, LEU534 HB: ARG120, TYR348, SER353, TYR385, MET522, GLY526, PHE529, SER530
Quercetin	−8.36	HI: VAL349, LEU352, LEU359, TRP387, ALA527; HB: ARG120, GLY526; PS: TYR355
Rutin	−13.87	HI: TYR115, VAL116, LEU352, TRP387, PHE518, VAL523; HB: HIS90, TYR355, ALA527, SER530; SB: ARG120

HI—hydrophobic interactions; HB—hydrogen bonds; HALB—halogen bond; PS—pi‐stacking; SB—salt bridges.

Regarding TRPV1, rutin again showed the most favorable interaction energy (−8.88 kcal/mol), followed by isoquercitrin (−7.29 kcal/mol), both surpassing capsazepine (−6.94 kcal/mol) (Table 6). Stabilization occurred primarily through hydrogen bonding with TYR511 and THR550, while isoquercitrin additionally interacted with ASN551 and GLU570.

**TABLE 6:** Interaction energy values between the TRPV1 receptor and each of the studied ligands, in kcal/mol, and the amino acid fragments of the active site that are interacting with them.

Ligand	Energy (kcal/mol)	Interactions
Capsazepine (+)	−6.94	HI: LEU515, LEU553, GLU570; HB: SER512, ARG557; HI: GLU513, ILE703
Gallic Acid	−6.20	HB: TYR495, ASP509, TYR511, GLU513, ARG557, GLN700; SB: ARG499; HI: LEU515, LEU553, ILE569, ILE573
Isoquercitrin	−7.29	HB: TYR511, THR550, ASN551, GLU570; HI: LEU512
Kaempferol‐3‐*O*‐rutinoside	−5.41	HB: TYR511, THR550; HI: LEU515
Quercetin	−5.13	HB: SER512, THR550, ARG557, GLU570
Rutin	−8.88	HB: TYR511, THR550

HI—hydrophobic interactions; HB—hydrogen bonds; HALB—halogen bond; PS—pi‐stacking; SB—salt bridges.

These findings corroborate the in vivo evidence obtained with EBM in the corneal nociception model and the reversal of its effect by capsazepine (Figure 4). Although rutin is present at lower concentrations than isoquercitrin and kaempferol‐3‐*O*‐rutinoside (Table 3), its predicted interaction with TRPA1 and TRPV1 remains pharmacologically relevant. Unlike the analyses involving COX‐2, the proposed participation of these receptors is supported by complementary evidence from both behavioral pharmacology and molecular docking, providing a more robust basis for suggesting that modulation of TRPA1 and TRPV1 contributes to the antinociceptive effects observed for the extract. Such affinity may compensate for its lower abundance, since compounds displaying strong receptor binding can exert biological effects even at reduced concentrations [23, 24]. Nevertheless, the biological activity of murici extracts cannot be attributed to a single constituent. The higher concentrations of isoquercitrin and kaempferol‐3‐*O*‐rutinoside likely contribute substantially to the observed effects through additive or synergistic interactions, a phenomenon widely recognized in plant‐derived mixtures [25, 26].

It should be emphasized that molecular docking provides only a theoretical estimate of ligand‐receptor affinity and does not account for pharmacokinetic parameters, metabolism, bioavailability, or synergistic interactions [27, 28]. Thus, the pronounced biological activity observed in vivo is likely the result of the combined action of multiple flavonoids. Likewise, although carrageenan‐induced edema is a well‐established experimental model for screening compounds with anti‐inflammatory potential, the present study did not evaluate inflammatory biomarkers such as cytokine production, COX‐2 expression, prostaglandin levels, NF‐κB activation, or leukocyte recruitment. Therefore, the molecular mechanisms underlying the edema‐reducing effect remain to be elucidated. Consequently, the proposed involvement of COX‐2 should be interpreted as a putative mechanism supported by computational predictions rather than direct experimental evidence.

Overall, the integration of phytochemical characterization, biological assays, and computational analyses demonstrates that murici extracts possess significant antioxidant, edema‐reducing, antiacetylcholinesterase, and antinociceptive activities. The behavioral pharmacology and molecular docking findings consistently support the involvement of TRPA1 in both extracts and TRPV1 in the bark extract in the observed antinociceptive effects. In contrast, the potential contribution of COX‐2 to the edema‐reducing effect remains hypothetical and requires confirmation through mechanistic studies involving inflammatory biomarkers. Collectively, these findings support murici as a promising source of multifunctional phytotherapeutic agents for the management of pain and inflammation.

## 3 Conclusions

The findings of this study demonstrate that the ethanolic extracts from the leaves (ELM) and bark (EBM) of *B. crassifolia* exert significant antinociceptive activity and reduce carrageenan‐induced abdominal edema in adult zebrafish. The involvement of TRPA1 and TRPV1 ion channels in the antinociceptive effect was supported by behavioral pharmacology assays, while HPLC analysis revealed kaempferol‐3‐*O*‐rutinoside as the major phenolic compound in the leaves and isoquercitrin as the predominant constituent in the bark, both flavonoids being widely recognized for their antioxidant, anti‐inflammatory, and neuroprotective properties.

Molecular docking analyses suggested that interactions with TRPA1, TRPV1, and COX‐2 may contribute to the observed pharmacological effects. However, although carrageenan‐induced edema is a well‐established model for screening anti‐inflammatory activity, the present study did not investigate inflammatory biomarkers. Therefore, additional mechanistic studies are required to confirm the molecular basis of the observed edema‐reducing activity and to validate the proposed involvement of COX‐2.

Among the evaluated compounds, rutin exhibited the most favorable binding profile, showing the lowest binding energies for TRPA1, COX‐2, and TRPV1. Its ability to establish multiple hydrogen bonds, together with stable hydrophobic and aromatic interactions, suggests high affinity for these targets. Notably, despite its lower abundance in the extracts, rutin may contribute to the biological activity because of its higher intrinsic binding affinity. Nevertheless, the pharmacological effects observed are unlikely to be attributable to a single constituent and are more likely to result from the combined action of multiple flavonoids acting through complementary and potentially synergistic mechanisms.

Finally, it is important to emphasize that molecular docking provides only a theoretical prediction of ligand–target interactions and does not account for factors such as bioavailability, metabolism, pharmacokinetics, or matrix effects. Overall, the integration of phytochemical characterization, behavioral pharmacology, and computational analyses supports the traditional use of *B. crassifolia* as a promising source of bioactive compounds with antinociceptive and edema‐reducing potential. Future studies should focus on isolating the principal active constituents, elucidating their synergistic interactions, and validating the molecular mechanisms underlying their anti‐inflammatory effects through targeted preclinical and, ultimately, clinical investigations.

## 4 Experimental Section

### 4.1 Obtaining the Material

The leaves and bark of murici were collected in February 2024 in Itapipoca, Ceará, Brazil. A specimen of this species is deposited in the Prisco Bezerra Herbarium (EAC) at the Federal University of Ceará (UFC), identified as *B. crassifolia* (L.) Kunth by Lígia Queiroz Matias, under registration number 67173. SisGen n° AFB0E62.

### 4.2 Preparation of Plant Extracts

The leaves and bark of murici (200 g) were collected, oven‐dried at 80°C, ground, and subjected to maceration with 70% ethanol in a 1:1 ethanol‐to‐material ratio at room temperature (25°C) for seven consecutive days, without renewal of the extracting solvent. The resulting extract was then filtered and concentrated using a rotary evaporator at 50°C. The solution was subsequently lyophilized, yielding the lyophilized hydroethanolic extract of murici leaves (ELM) and the lyophilized hydroethanolic extract of murici bark (EBM).

### 4.3 High‐Performance Liquid Chromatography (HPLC) with Diode Array Detector (DAD)

To identify phenolic compounds, a methanolic solution of each sample (20 µL/mL) was injected into the HPLC system. Standards were obtained from Sigma, and analytical‐grade solvents were used for extraction, while HPLC‐grade solvents were used in the analyses. Samples and solutions were filtered through nylon membranes (0.45 µm and 0.22 µm) before analysis, which was performed in triplicate. HPLC‐DAD analysis was conducted using a Shimadzu system with a Shim‐pack ODS GOLD reversed‐phase column. The mobile phases were acetonitrile and Milli‐Q water acidified to pH 2.8, using a gradient: 0–15 min (20:80 v/v), 17–25 min (40:60 v/v), and 25–40 min (20:80 v/v). The flow rate was 1.0 mL/min, with a 20 µL injection volume and detection at 350 nm. Peaks were confirmed by retention time and DAD spectra (200–400 nm).

### 4.4 Quantification of Total Phenols, Flavonoids, and Tannins

The determination of total phenolic content was performed by visible spectrophotometry using the Folin–Ciocalteu reagent [29]. For the quantification of flavonoids, a 2.5% aluminum chloride (AlCl_3_) reagent was used [30]. The determination of total tannin content was carried out using visible region spectroscopy with the Folin‐Denis reagent [31]. The Thermo Genesis 10S UV–vis spectrophotometer was used for the test.

### 4.5 Biological Activity In Vitro

The antioxidant activity was evaluated using the DPPH (2,2‐diphenyl‐1‐picrylhydrazyl) method [32], and by the ABTS (2,2′‐azino‐bis(3‐ethylbenzothiazoline‐6‐sulfonic acid)) method, both with modifications [33]. Both tests were performed in a 96‐well flat‐bottom microplate using a BioTek ELX 800 ELISA reader. The inhibitory activity of the acetylcholinesterase (AChE) enzyme was measured in flat‐bottom 96‐well plates using a BIOTEK ELISA reader, model ELX 800, with the “Gen5 V2.04.11” software [34, 35]. All samples were analyzed in triplicate.

### 4.6 Zebrafish

Adult zebrafish (*Danio rerio*) (ZFa), wild type, of both sexes, aged 90 days, measuring 3.5 ± 0.5 cm in length and weighing 0.4 ± 0.1 g, were obtained from Agroquímica: Comércio de Produtos Veterinários LTDA, a supplier in Fortaleza (Ceará, Brazil). Groups of 40–50 fish were acclimated in 9 L glass aquaria at room temperature (26 ± 2°C) for 24 h, containing dechlorinated water (ProtecPlus) and air pumps with submerged filters, maintained at 25°C and pH 7.0, under a 14:10 h light/dark cycle. The fish were fed ad libitum 24 h before the experiments. All experimental procedures were approved by the Ethics Committee on Animal Use of the State University of Ceará (CEUA‐UECE), under protocol No. 04009489/2023.

#### 4.6.1 General *P*rotocol

On the day of the experiments, adult zebrafish (ZFa) were randomly assigned to the experimental groups. The test samples were dissolved in 3% dimethyl sulfoxide (DMSO), which was used as the vehicle for all treatments. The fish were then transferred onto a moist sponge and treated with the test or control samples via intraperitoneal (i.p.) or oral (p.o.) administration. They were then individually placed in glass beakers (250 mL) containing 150 mL of aquarium water for recovery. For intraperitoneal (i.p.) treatments, insulin syringes (0.5 mL; UltraFine BD) fitted with 30G needles were used. For oral (p.o.) treatments, a variable‐volume 20 µL micropipette was used. The animals’ behavior was recorded by calibrated and blinded observers [36, 37].

#### 4.6.2 Acute *T*oxicity 96 h

The acute toxicity study was carried out on adult zebrafish in accordance with the guidelines of the Organization for Economic Cooperation and Development (OECD) and the Standard Development Method [38] to determine the 96 h LC_50_. The animals (*n* = 6 per group) were treated orally (p.o.) with 20 µL of the test sample (0.01, 0.1, 1.0, 10, or 100 mg/mL) or vehicle (3% DMSO; control). After treatment, zebrafish mortality was assessed every 24 h. After 96 h, the number of dead fish in each group was recorded, and the lethal concentration capable of killing 50% of the animals (LC_50_) was determined using the Trimmed Spearman–Karber statistical method with a 95% confidence interval [39].

#### 4.6.3 Open Field Test

The Field Test was carried out to evaluate the sedative effect or muscle relaxation in animals treated with the test samples prior to exposure to AlCl_3_ (100 µg/L; pH 5.8). The animals (*n* = 6 per group) were treated orally (p.o.) with 20 µL of the test sample (0.01, 0.1, 1.0, 10, or 100 mg/mL) or vehicle (3% DMSO; control). An additional group (*n* = 6) without treatment was used as the naive control. One hour after oral administration, the animals were placed in Petri dishes (100 × 15 mm) containing the same aquarium water, marked with four quadrants, and the neuroprotective effect was assessed based on the natural locomotor activity of the zebrafish, quantified by counting the number of line crossings (LC) during a 0–5 minute observation period [36, 40].

#### 4.6.4 Behavioral Antinociceptive Activity

The concentrations of the test sample solutions that did not alter the locomotor activity of the animals were selected. The concentrations of the noxious agents: formalin (0.1%; 5.0 µL; TRPA1 agonist), hypertonic saline (NaCl 5M; 3.0 µL; TRPV1 agonist), or LPS (0.0625 mg/mL; 3.0 µL; TRPA1 agonist) and antagonists capsazepine (TRPV1 antagonist; 0.5 mg/mL; 5 µL) and HC‐030031 (TRPA1 antagonist; 0.1 mg/mL; i.p.) as well as the duration of nociceptive response analysis [36, 41, 42, 43, 44].

For behavioral analyses, after treatment with nociceptive agents or antagonists, the animals were individually placed in glass Petri dishes (100 × 15 mm) divided into quadrants, and the nociceptive or antinociceptive response was quantified in terms of locomotor activity (LA) or line crossings (LC) performed during a specific observation period for each model described below.

#### 4.6.5 Formalin‐Induced Nociceptive Behavior

Adult zebrafish were pretreated (20 µL; p.o.) with the test sample (0.01, 0.1 or 1.0 mg/mL), morphine (Mor; positive control; 0.2 mg/mL; i.p.), or vehicle (control, 3% DMSO) 30 min before intramuscular injection of formalin (0.1%; 5.0 µL; TRPA1 agonist) into the tail of the animals (*n* = 6 per group). A naive (untreated) group was also included. The antinociceptive effect was characterized by an increase in locomotor activity (LA) during the neurogenic phase (0–5 min) and the inflammatory phase (15–30 min) [36].

#### 4.6.6 Corneal Nociceptive Behavior Induced by Hypertonic Saline

Corneal nociception was induced using a hypertonic saline solution (TRPV1 agonist; NaCl 5.0 M; 5.0 µL) applied to the right eye of the animals (*n* = 6 per group), 1 h after pretreatment with the test sample (0.01, 0.1 or 1.0 mg/mL), morphine (Mor; positive control; 0.4 mg/mL; 20 µL; i.p.), or vehicle (negative control; 3% DMSO; 20 µL; p.o.). An untreated (naive) group was also included. The corneal antinociceptive effect was characterized by an increase in locomotor activity (LA) during the 0–5 min observation period [41].

This test was chosen to evaluate the possible involvement of TRPV1 channels (see Results). Therefore, in a subsequent experiment, the animals (*n* = 6 per group) were pretreated intraperitoneally with capsazepine (TRPV1 antagonist; 0.5 mg/mL; 20 µL; i.p.) 15 min before the oral administration of the lowest effective dose of the test sample.

#### 4.6.7 Lipopolysaccharide (LPS)‐Induced Abdominal Nociceptive Behavior

Abdominal nociception was induced using a lipopolysaccharide solution isolated from *Escherichia coli* (LPS; TRPA1 agonist; 0.0625 mg/mL; 3.0 µL), administered intraperitoneally (i.p.) into the abdomen of the animals (*n* = 6 per group), 1 h after pretreatment with the test sample (0.01, 0.1 or 1.0 mg/mL), morphine (Mor; positive control; 0.2 mg/mL; 5.0 µL; i.p.), or vehicle (negative control; 3% DMSO; 20 µL; p.o.). An untreated (naive) group was also included. c

### 4.7 Computational Study

#### 4.7.1 Protein Preparation

The structure of the TRPA1 ion channel receptor (PDB: 3J9P) [46], obtained with a resolution of 4.24 Å and that of TRPV1 (PDB: 5IS0) [47], complexed with the capsazepine molecule, obtained with a resolution of 3.43 Å, both determined by electron microscopy and COX2 (PDB: 3NT1) [48], complexed with the naproxen molecule, obtained at a resolution of 1.73 Å and determined by X‐ray diffraction, were retrieved from the RCSB Protein Data Bank. Subsequently, all water molecules and other existing compounds were removed. Polar hydrogen atoms and Kollman atomic charges were then added.

#### 4.7.2 Preparation of Binders

The two‐dimensional structures of the compounds HC‐030031, diclofenac, capsazepine, gallic acid, isoquercitrin, kaempferol‐3‐*O*‐rutinoside, quercetin, and rutin were drawn using the free version of the ChemSketch program. Subsequently, their 3D structures were prepared using the free version of the 3D Viewer software.

#### 4.7.3 Molecular Docking

Molecular docking was performed to predict the orientation of the complexes formed between the ligands HC‐030031, diclofenac, capsazepine, gallic acid, isoquercitrin, kaempferol‐3‐*O*‐rutinoside, quercetin, and rutin and the receptors TRPA1, COX‐2, and TRPV1, as well as to obtain the binding energy to estimate the interaction strength [49]. The HC‐030031 molecule was used as a reference for TRPA1, diclofenac was used as a reference for COX‐2, and capsazepine was used as a reference for TRPV1. The calculations took into account hydrogen bond formation, attractive van der Waals interactions, electrostatic interactions, and attractive hydrophobic interactions [50]. AutoDock 4.2 software it was used to perform automated dockings in order to identify the possible binding modes between the receptors and the studied ligands [51]. During the calculation procedure, all receptors were kept rigid, while all ligands were considered flexible. The Lamarckian genetic algorithm was chosen as the search parameter. The number of runs for each docking was set to 50, allowing each ligand to adopt 50 different conformations and bind freely to any position of the receptor within the grid box dimensions. The grid box position was consistent with that extracted from the crystal structure and was set to 60 × 60 × 60 Å. The grid point spacing was set to 0.375 Å. The analysis of the obtained results was performed using the AutoDock Tools program and subsequently visualized in the Chimera software [52].

## Author Contributions

The authors declare individual contributions to the article as follows: data collection, analysis and the preparation of the manuscript: L.S.F.: conceptualization; writing – original draft and writing – review and editing; S.I.C.G.B.: methodology; M.R.C.d.O.: methodology; G.A.d.N.: methodology; S.A.A.R.S.: methodology; H.M.I.: methodology; F.E.A.M.: methodology; A.R.C.: methodology and funding acquisition; S.M.d.M.: conceptualization; funding acquisition and writing – review and editing. The completed manuscript underwent comprehensive review by all authors, resulting in approval for publication. All authors have read and agreed to the published version of the manuscript.

## Conflicts of Interest

The authors declare no conflicts of interest.

## Data Availability

The data that support the findings of this study are available from the corresponding author upon reasonable request.
